# Noninvasive estimation of internal spinal alignment in patients with adolescent idiopathic scoliosis using PCdare and back shape asymmetry

**DOI:** 10.1038/s41598-025-95902-1

**Published:** 2025-03-29

**Authors:** Mirko Kaiser, Emily McLaughlin, Martin Bertsch, Christoph J. Laux, Mazda Farshad, Tobia Brusa, Volker M. Koch, William R. Taylor, Saša Ćuković

**Affiliations:** 1 Laboratory for Movement Biomechanics, ETH Zurich, Zurich, Switzerland; 2 University Spine Center Zurich, Balgrist University Hospital, University of Zurich, Zurich, Switzerland; 3 Biomedical Engineering Laboratory, Bern University of Applied Sciences, Biel, Switzerland

**Keywords:** Adolescent idiopathic scoliosis, Noninvasive assessment, 3D surface scanning, Asymmetry map, Radiography, Translational research, Radiography, Software, Imaging and sensing, Three-dimensional imaging, Skeleton, Bone quality and biomechanics

## Abstract

Optical 3D surface scanning is used increasingly to assess spinal deformity in patients with adolescent idiopathic scoliosis (AIS), largely because it avoids additional radiation burden. However, such approaches generally underestimate the extent of the abnormality. Improving the accuracy of such estimates requires a deeper understanding of AIS and its effect on back shape. We present a unique platform with publicly available code that contains noninvasive and nonionizing approaches to estimating the Cobb angle of the primary curve, called primary Cobb angle (pCA) and internal spinal alignment (ISL) in patients with AIS. Our approaches use asymmetries of the back shape during upright standing, the Adam’s forward bending test, bending forward, and lateral bending. The results have revealed strong (0.75 [0.53, 0.87]) to excellent (0.91 [0.81, 0.96]) correlations [95% confidence interval] and a median pairwise absolute error (IQR) of 3.4° (6.8°) between the estimated pCAs and clinical gold-standard assessments in 30 patients. The correlations (IQR) between estimated shape of ISLs and their references were very strong (0.87 (0.24)) to excellent (0.94 (0.03)), and the median root mean square error (IQR) between estimated and reference ISL was 6.9 mm (3.3 mm). These results indicate confidence both in the use of 3D scanning using a “back-shape-to-spine” approach and in the establishment of optical 3D surface scanning approaches for scoliosis screening and monitoring in clinical practice.

## Introduction

Adolescent idiopathic scoliosis (AIS) is by far the most common deformity of the spine and typically occurs in teenagers. If left untreated, advanced deformities can induce cardiopulmonary impairment, cosmetic alterations, and discomfort^1^. The current clinical gold standard for assessing AIS is radiography. Clinical monitoring can expose patients with AIS to extensive radiographic imaging up to approximately every 6 months, resulting in a significant radiation burden in young individuals^2^. Consequently, several studies have reported that the incidence of radiation-induced cancer types in patients with AIS is five times higher than in individuals without AIS^3,4^. As a result, optical 3D surface scanning is increasingly investigated and used in medical applications as a noninvasive and nonionizing alternative for screening and monitoring, mostly in young individuals^5–11^.

Scoliosis is defined as a lateral curvature of the spine with a Cobb angle of primary curve, called primary Cobb angle (pCA), of 10 degrees or greater; the Cobb angle is the frontal angle between the endplates of the two most tilted vertebrae measured on radiographs typically acquired in a static, upright standing position. However, correlations between pCAs estimated solely from the 3D back surface in “back-shape-to-spine” approaches used in clinical practice and pCAs obtained from radiographs remain only moderate^5,12,13^.

Current research investigates asymmetry approaches, in which asymmetries in the shape of the back are leveraged to estimate pCAs in patients with AIS, and has shown promising results^7,14^. However, these approaches have several limitations: they are primarily restricted to static standing upright or the Adam’s forward bending test and estimate only Cobb angles without providing insights into curvature location and shape. Furthermore, code is not shared which limits reproducibility.

To further improve these back-shape-to-spine approaches, better understanding is required of the relationship between back shape and spinal alignment, in this work using the line through the centroids of vertebral bodies (internal spinal alignment, ISL). To achieve this goal, estimations are required not only of pCAs but also of the ISL while standing upright. Unlike a single Cobb angle value, the ISL enables a more detailed characterization of the shape of the spinal curvature. This is particularly important for dynamic scoliosis evaluation.

In this paper, we present a unique platform with publicly available code (see Data Availability Statement) for noninvasive and nonionizing approaches to estimating pCA and ISL in patients with AIS. The approaches involve back shape asymmetries captured with optical 3D scanning during upright standing, Adam’s forward bending test, bending forward, and lateral bending.

## Methods

**Fig. 1:**
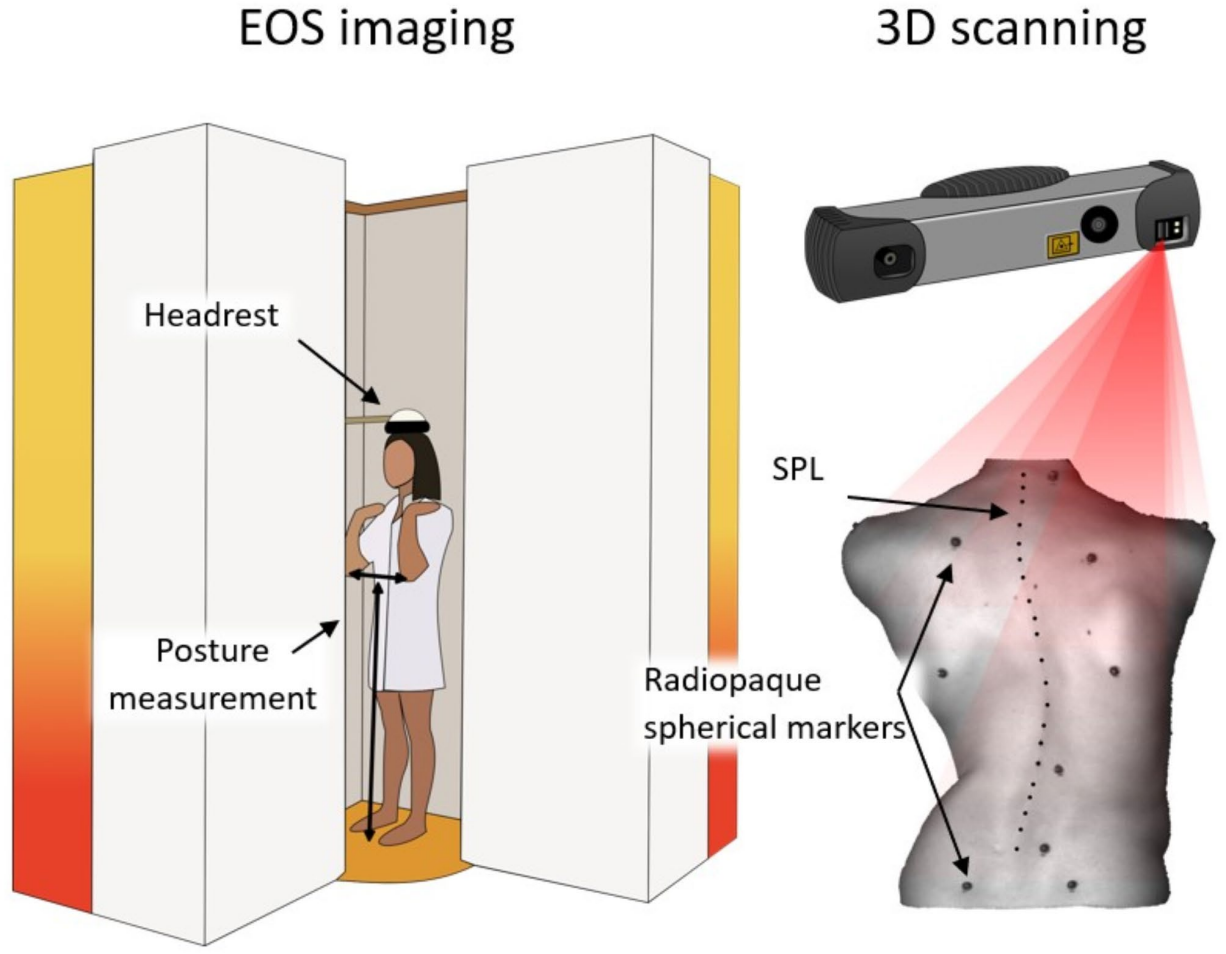
Left) EOS imaging: The headrest was used, and the distances between elbows and between elbows and floor were measured to record the posture. Right) Optical 3D scanning of the patient’s back with nine radiopaque spherical markers on anatomical landmarks (C7, left and right medial end of the scapular spine (MSS), left and right inferior scapular angle (ISA), T12, L5, and left and right posterior superior iliac spine (PSIS)) and pen markings from C7 to L5 along the spinous process line (SPL).

**Fig. 2:**
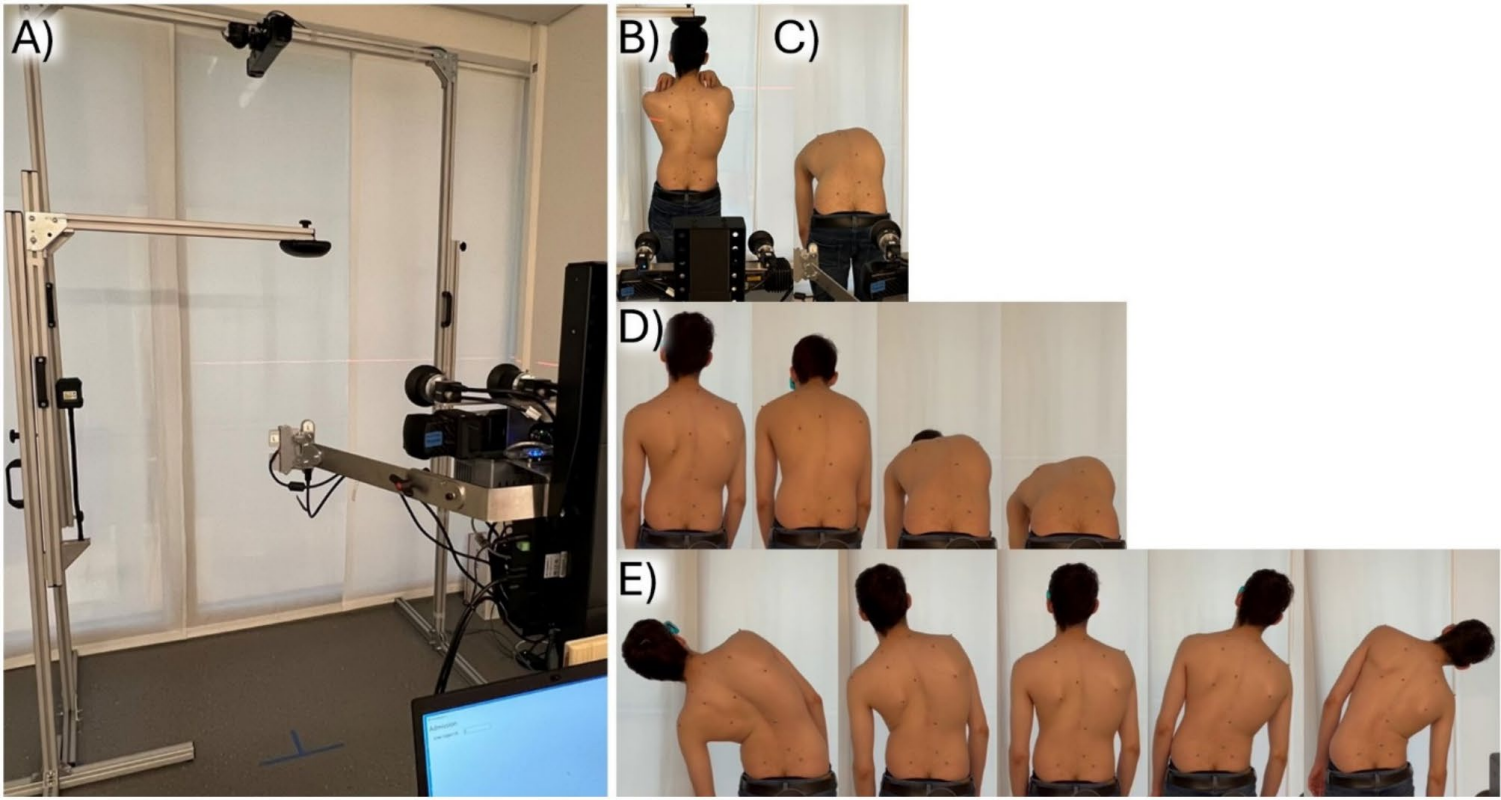
(**A**) Setup at the academic spine center with headrest replication and cameras from above and behind; illustration of postures for (**B**) upright standing and (**C**) Adam’s forward bending test, and movements for (**D**) bending forward and (**E**) lateral bending.

Data from 30 adolescents and young adults with scoliosis (with mean age ± std of 18 years ± 4 years, Cobb angle of primary curve (pCA) of 28° ± 12°, BMI of 20 kg/m^2^ ± 3 kg/m^2^, 21 females and 9 males) were collected at an academic spine center. The patients were each equipped with nine radiopaque spherical markers on anatomical landmarks (C7, left and right medial end of the scapular spine (MSS), left and right inferior scapular angle (ISA), T12, L5, and left and right posterior superior iliac spine (PSIS)), and pen markings were applied along the 17 spinous processes from C7 to L5 (Fig. 1). The patients underwent biplanar radiography (EOS imaging, Paris, France) followed by optical 3D surface scanning with the Photoneo MotionCam-3D (Photoneo s.r.o, Bratislava, Slovakia) in upright standing posture, Adam’s forward bending test, dynamic bending forward, and dynamic lateral bending (Fig. 2). For upright standing, the posture was replicated with posture measurements during the EOS imaging. For the Adam’s forward bending test, the patient was asked to remain in a position where the angle of trunk rotation was visually at its maximum^15^. For bending forward and lateral bending, the patient was asked to bend as far forward, to the left, and to the right as possible, and the sequence was captured dynamically with 5 Hz.

**Fig. 3:**
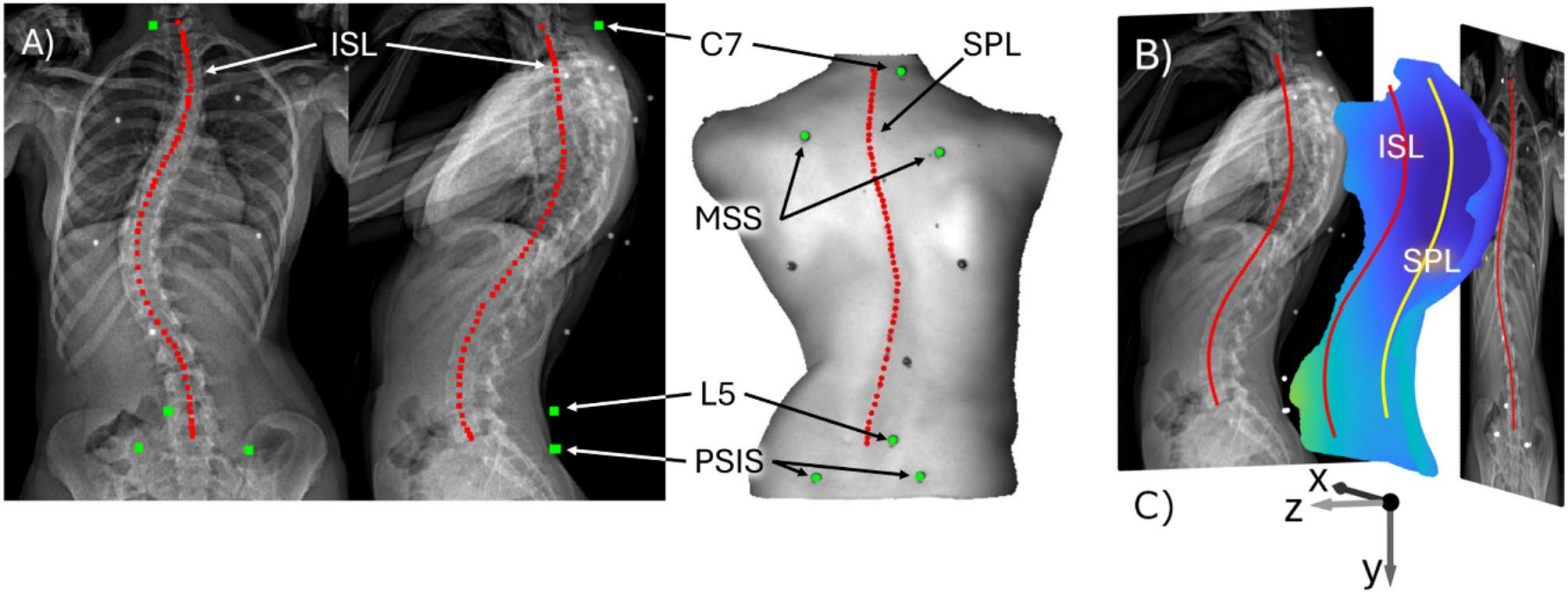
(**A**) Manual selection of anatomical landmarks C7, MSS, L5, PSIS, SPL, and ISL with (**B**) automatic registration of the 3D back surface scan with the biplanar radiographic images in PCdare, and (**C**) coordinate system (x, y, z).

The data from all 30 patients were processed with the PCdare software^16^. Six marker locations were manually selected with PCdare for C7, left and right medial end of the scapular spine (MSS), L5, and left and right posterior superior iliac spine (PSIS) (Fig. 3A, green). Furthermore, the pen markings along all spinous processes (spinous process line, SPL) and the line through the centroids of vertebral bodies (internal spinal alignment, ISL) were manually digitalized as regularly sampled points from C7 to L5 and used as reference (Fig. 3A, red). The 3D surface scans were then rigidly registered automatically with the biplanar radiographs (Fig. 3B).

### Method for estimating the spinous process line

The SPLs for lateral bending were estimated from C7 to L5 with a U-Net^17^ trained on 664 depth map images sampled uniformly from the dynamic captures. The depth map images were generated by projecting the 3D surface scans on to the coronal plane. The trained model was then used to estimate the SPL for all optical 3D surface scans during lateral bending (3809 images). The performance of the trained model was evaluated with the root mean square error (RMSE) and interquartile range (IQR) between estimated and reference SPL. Due to the limited number of images for scans other than lateral bending, the manual SPLs were used for upright standing, Adam’s forward bending test, and bending forward.

### Method for estimating the Cobb angle of the primary curve

**Fig. 4:**
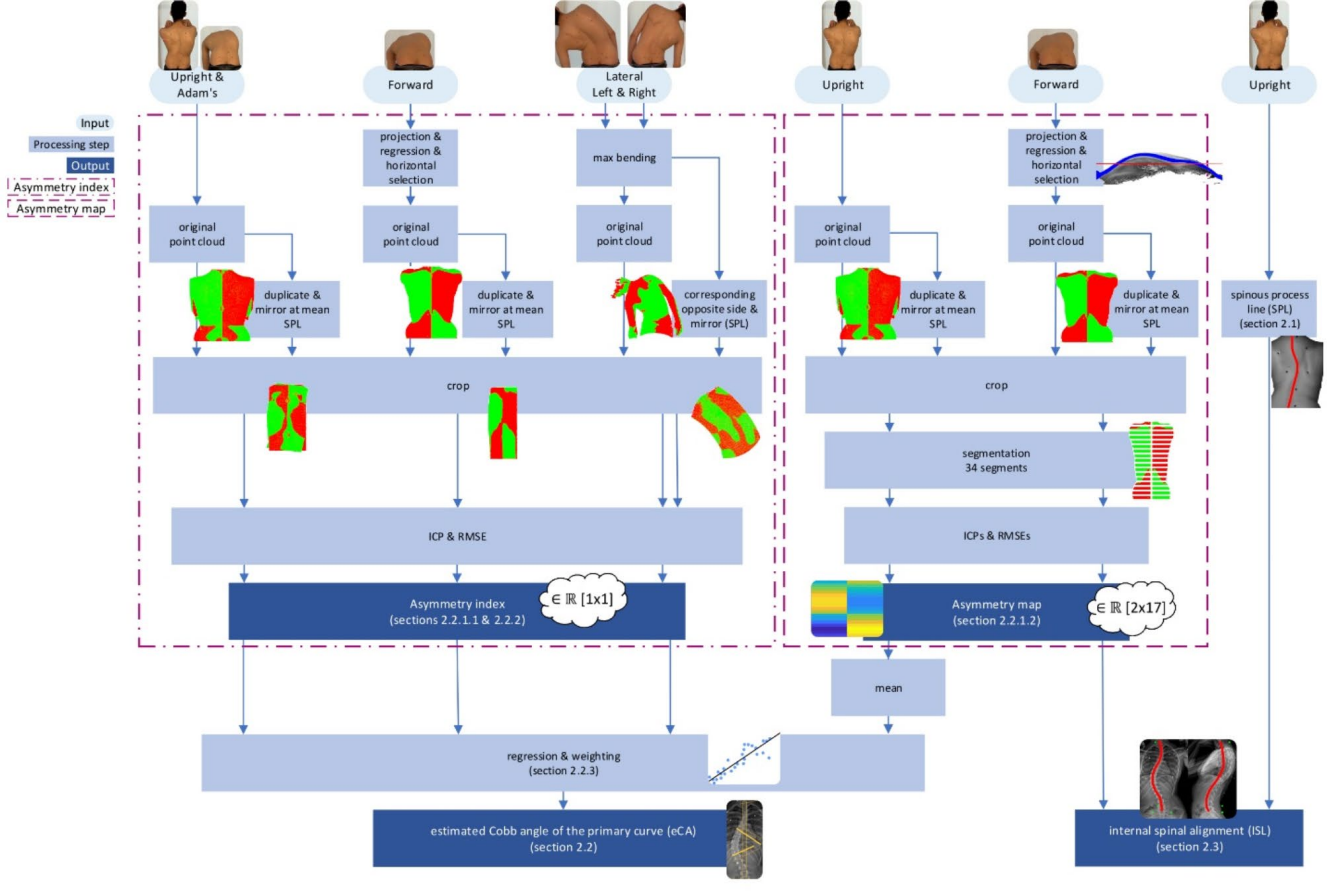
Graphical overview of asymmetry index, asymmetry map, and SPL approaches applied to upright standing, Adam’s forward bending test, bending forward, and lateral bending to estimate rCA and ISL.

The pCA in an upright standing position was estimated with asymmetry index (“Asymmetry index” and “Asymmetry index for lateral bending” sections) and asymmetry map (“Asymmetry map” section) approaches^14,18^ derived from optical 3D surface scans of the back shape in different postures and movements (Table 1, Fig. 4).

**Table 1:** Overview of asymmetry index, asymmetry map, and SPL approaches applied to upright standing, Adam’s forward bending test, bending forward, and lateral bending to estimate rCA and ISL in an upright standing position.

	Cobb angle (upright standing)				ISL (upright standing)	
	Upright	Adam’s	Forward	Lateral	Upright	Forward
Asymmetry index	✓	✓	✓	✓	╳	╳
Asymmetry map	✓	╳	╳	╳	╳	✓
SPL	╳	╳	╳	╳	✓	╳

The asymmetry index approach (“Asymmetry index” and “Asymmetry index for lateral bending” sections) was applied to upright standing, Adam’s forward bending test, bending forward, and lateral bending to estimate the radiographic Cobb angle of the primary curve (rCA) in an upright standing position. The asymmetry map approach (“Asymmetry map” section) was also applied to upright standing to estimate the rCA. Furthermore, the ISL in an upright standing position was estimated using the SPL from upright standing and the asymmetry map approach applied to bending forward (“Method for estimating internal spinal alignment” section).

#### Asymmetry for upright standing, Adam’s forward bending test, and bending forward

##### Asymmetry index

**Fig. 5:**
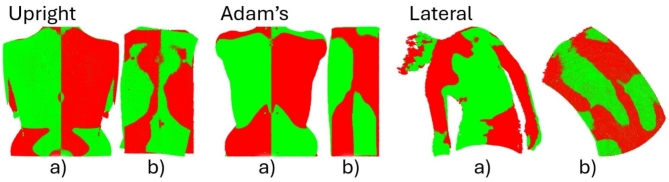
(**a**) Original (red) and mirrored (green) point clouds and (**b**) cropped (red) and mirrored cropped (green) point clouds after applying an iterative closest point algorithm (ICP) for upright standing, Adam’s forward bending test, and lateral bending. After mirroring, cropping, and ICP, the asymmetry index was computed as the RMSE value between cropped and mirrored cropped point clouds. This asymmetry index was then used to estimate the primary Cobb angle.

To estimate the pCA in an upright standing position using an asymmetry index, one optical 3D surface scan was selected as point cloud for each posture and movement. Only single static point clouds were obtained for upright standing and the Adam’s forward bending test. To obtain the point cloud that represents the nearest alignment to horizontal for the bending forward movement, each point cloud in the dynamically captured sequence was projected onto the sagittal plane to extract the lateral inclination. The point cloud selected was the one in which the linear regression of all points was closest to zero and thus had the lowest inclination. This original point cloud was then duplicated and mirrored axis-symmetrically around the y-axis (Fig. 3C) at the mean location of the manual SPL (Fig. 5). The manual SPL was also used to limit the point clouds from C7 to L5 in caudal and cranial directions (y-axis, Fig. 3C).

To remove border artefacts such as arms, the 0.1 and 0.9 quantiles of all points in medial and lateral direction (x-axis) were calculated, and points further than 75% of the minimum distance between mean SPL location and quantiles were cropped.

An iterative closest point algorithm (ICP, pcregistericp MATLAB function) was then applied to minimize the RMSE between cropped point clouds. The asymmetry index was then calculated as the RMSE between nearest neighbors for both the cropped and mirrored cropped point clouds.

##### Asymmetry map

**Fig. 6:**
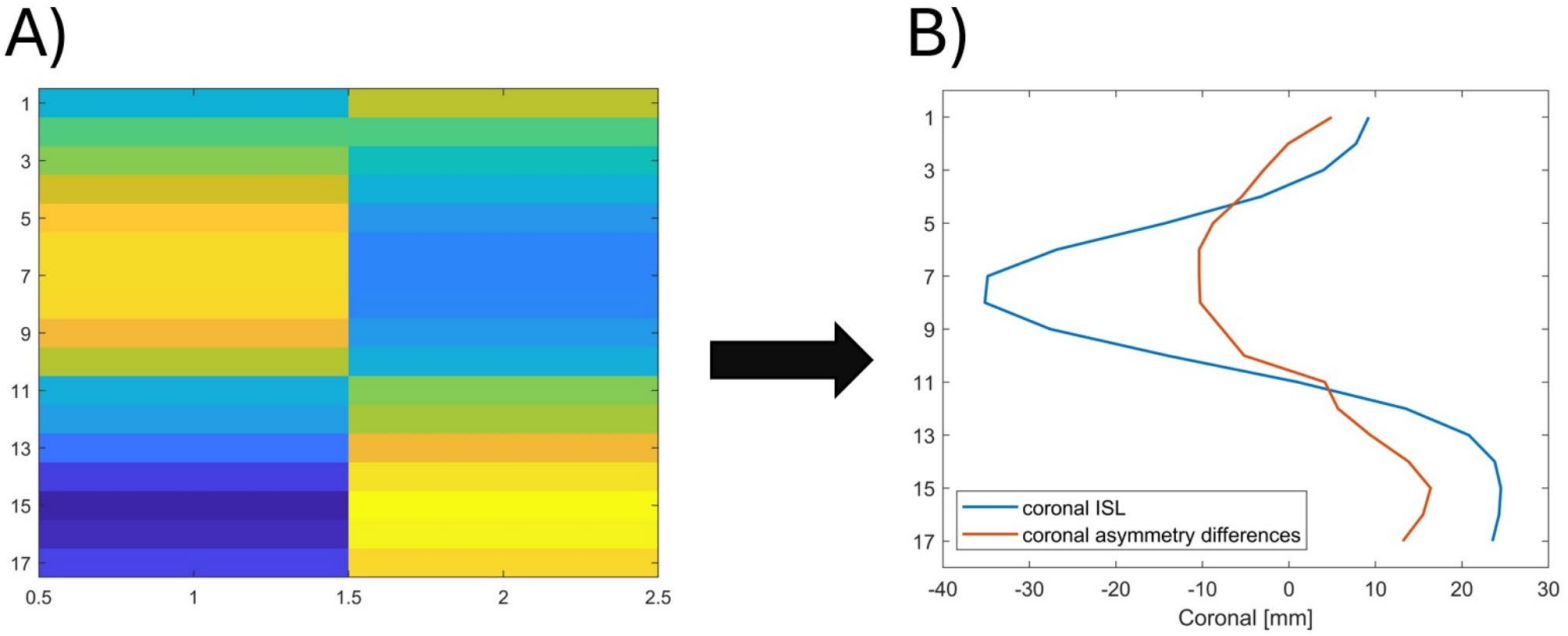
(**A**) Example of an asymmetry map consisting of 2 × 17 segments in which the colors indicate the RMSE values calculated between each cropped segment and its mirrored counterpart. The asymmetry map was constructed by first mirroring and cropping the point cloud (Fig. 5), then dividing both the original and mirrored cropped point clouds into 2 × 17 segments along the cranial-caudal axis, followed by applying an iterative closest point algorithm to each segment pair to compute the RMSE values. (**B**) Coronal asymmetry differences computed as the difference between the RMSE values of left and right segment pairs were used to estimate the coronal curvature of the ISL.

The pCA in an upright standing position was estimated with an asymmetry map constructed from the point cloud obtained during upright standing (“Asymmetry index” section). After mirroring the point cloud at the mean location of the SPL, the cropped and the mirrored cropped point clouds (Fig. 5b) were divided into 17 regions uniformly distributed in caudal and cranial directions. Each of the 17 regions was then further divided into two segments left and right of the mean SPL. An ICP algorithm was then applied to each pair of the 34 cropped and 34 mirrored cropped segments individually. The resulting RMSEs for each segment pair were then used as an asymmetry map with 34 asymmetry indices (Fig. 6A). The overall asymmetry map index was then calculated using the mean of all 17 segments from the right side.

#### Asymmetry index for lateral bending

The asymmetry index for lateral bending was calculated by first finding the maximum bending to the left and right with the estimated SPL (“Method for estimating the spinous process line” section). Maximum bending was defined as the lowest horizontal inclination in a linear fit between the mean values of the upper and lower 50th percentiles. For the left maximum bending, the corresponding SPL was calculated from lateral bending to the right. To do so, all SPLs from the whole lateral bending sequence to the right were mirrored axis-symmetrically around the y-axis in the medial direction. Then, the SPL with the smallest RMSE between nearest neighbors was calculated. The corresponding point cloud of the 3D back shape was then also mirrored in medial direction (Fig. 5 Lateral). All points from both the left bending and corresponding mirrored right bending point cloud that were outside a certain radius from the mean SPL were then removed. Half the distance between left and right MSS was used as the radius. Then, an ICP algorithm was run, and the RMSE between both point clouds was calculated. The same procedure was applied for the right maximum bending. The mean value of the two individual RMSEs was then used as an asymmetry index.

#### Regression and weighting

A linear regression (fitlm MATLAB function) between asymmetry indices and rCA was used to estimate the pCAs in an upright standing position from the asymmetry indices for each posture and movement. To obtain a more robust overall estimate of the pCAs (eCA), the individually estimated Cobb angles were then weighted with factors: 4 for bending forward, 3 for Adam’s forward bending test, 2 for upright standing asymmetry map approach, 1 for upright standing asymmetry index approach, and 1 for lateral bending.

#### Comparison with clinical gold standard

The Pearson correlation coefficient (corrcoef MATLAB function) was calculated between asymmetry indices and rCAs in an upright standing position manually annotated by a board-certified spine surgeon in PACS (MERLIN Diagnostic Workcenter, Version 7.1, Phönix-PACS GmbH, Freiburg im Breisgau, Germany). Furthermore, the Pearson correlation coefficient was calculated between eCAs and rCAs, and median pairwise absolute error (MAE) and interquartile range (IQR) between eCAs and rCAs were used to evaluate estimation accuracy.

### Method for estimating internal spinal alignment

The line through the centroids of vertebral bodies (ISL) was estimated for upright standing with the manual SPL from upright standing and the asymmetry map for bending forward (“Asymmetry map” section, Table 1). The manual SPL was used as initial estimate for the ISL, and the coronal curvature of the ISL was adjusted with the coronal asymmetry differences between left and right RMSEs from the asymmetry map for bending forward (Fig. 6).

Pearson correlation coefficients between coronal asymmetry differences and coronal reference ISL and median RMSE and IQR between estimated and reference ISL were used to evaluate the estimation accuracy. To estimate the pCA, the absolute position of the ISL is not relevant; therefore, we also evaluated Pearson correlation coefficients for the coronal shape, when we allowed the estimated ISL to be shifted two segments up or down. This indicates how well the overall coronal shape correlates between estimated ISL and reference ISL irrespective of a limited vertical offset.

## Results

### Estimation of the spinous process line

The median RMSE between estimated SPL and reference SPL was 1.9 mm with an IQR of 1.8 mm for the training data, which contained 664 images of lateral bending.

### Estimation of Cobb angle of the primary curve

**Fig. 7:**
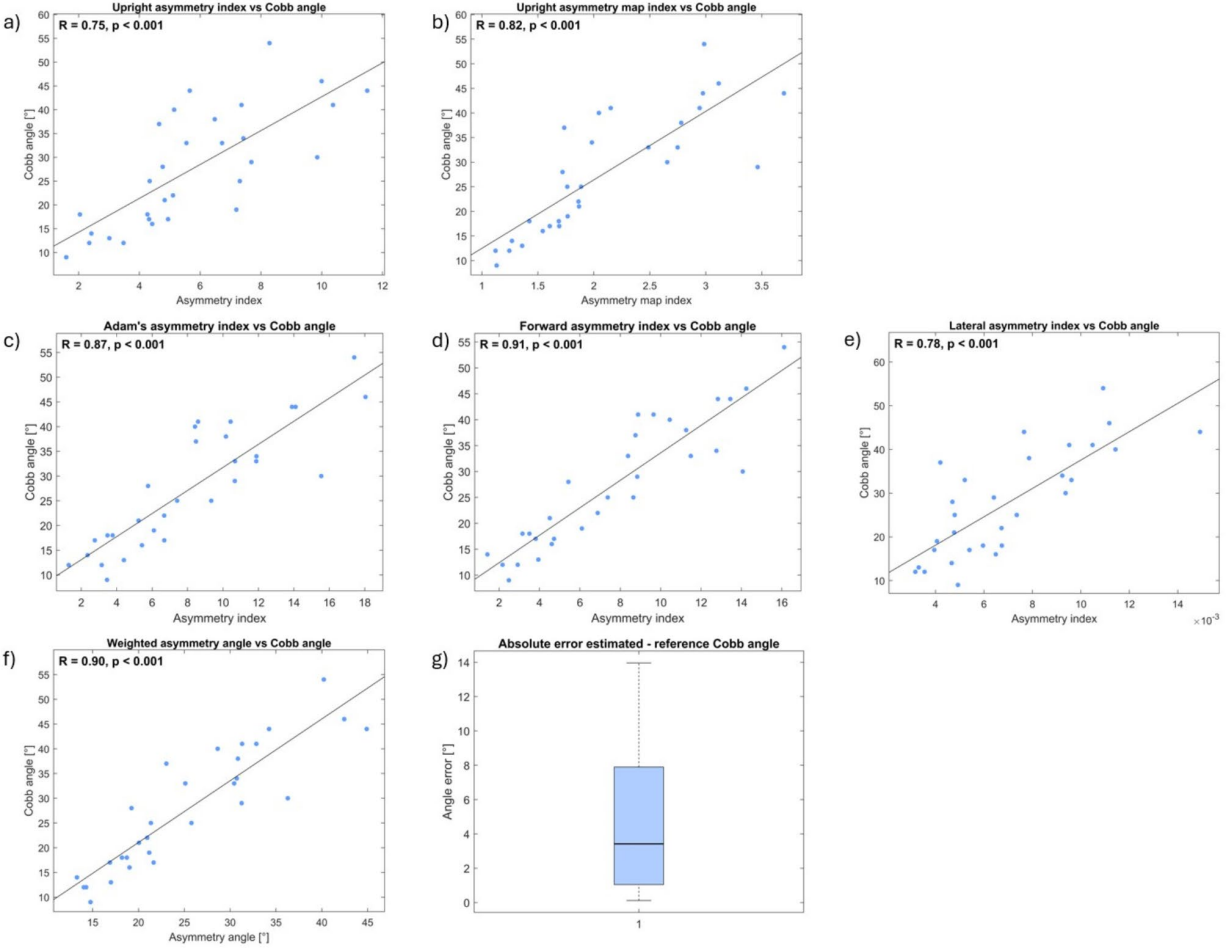
Pearson correlation coefficient between rCA and (**a**) upright asymmetry index, (**b**) upright asymmetry map index, (**c**) Adam’s forward bending test asymmetry index, (**d**) bending forward asymmetry index, (**e**) lateral bending asymmetry index, and (**f**) eCA. (**g**) Boxplot for pairwise absolute error between rCA and eCA.

The Pearson correlation coefficient (95% confidence interval) between asymmetry index and radiographic Cobb angle of the primary curve (rCA) for upright standing, Adam’s forward bending test, bending forward, and lateral bending for 30 patients ranged between 0.75 and 0.91 ([0.53, 0.87] and [0.81, 0.96]). The correlation between the overall estimate of the pCA (eCA) and rCA was 0.90 ([0.79, 0.95]), and the median pairwise absolute error (IQR) between eCA and rCA was 3.4° (6.8°) (Fig. 7).

### Estimation of internal spinal alignment

The median Pearson correlation coefficients and IQRs between coronal asymmetry difference and coronal reference ISL and between the shape of the coronal asymmetry difference and coronal reference ISL are shown in Table 2.

**Table 2:** Median Pearson correlation coefficients (PCC) and interquartile ranges (IQR) between coronal asymmetry differences and coronal curvature of the reference ISL (first row) and shape of coronal curvature of the reference ISL (second row). Columns: All 30 patients, all patients with confirmed AIS (pCA ≥ 10°), patients with moderate AIS (pCA ≥ 25°), and patients with severe AIS (pCA ≥ 45°).

	All patients (n = 30)	AIS (n = 29)	Moderate AIS (n = 17)	Severe AIS (n = 2)
Median PCC (IQR)	0.67 (0.36)	0.68 (0.35)	0.78 (0.24)	0.86 (0.07)
Median PCC (IQR) shape	0.87 (0.24)	0.87 (0.24)	0.92 (0.10)	0.94 (0.03)

**Fig. 8:**
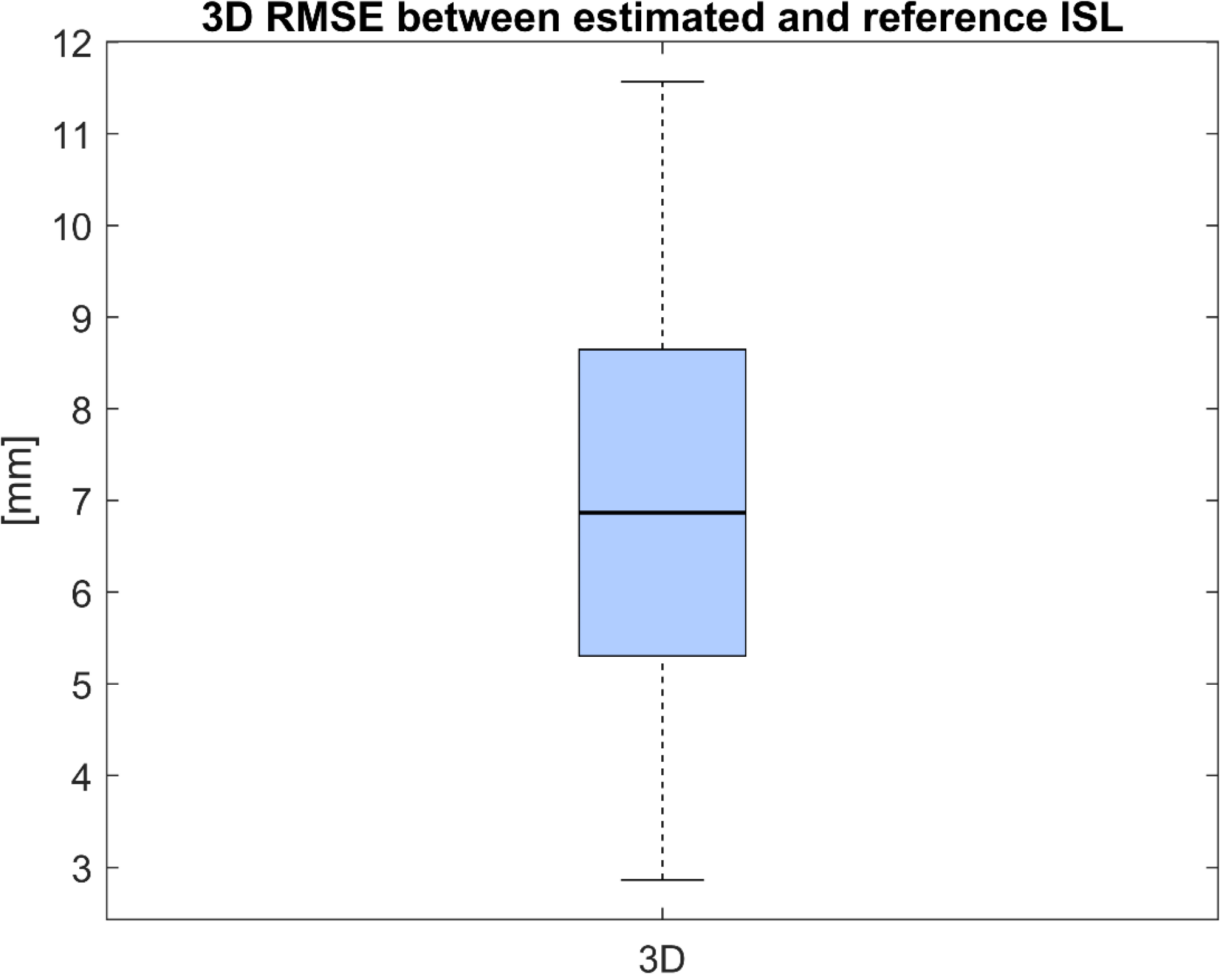
Boxplot of RMSEs in mm between estimated and reference ISLs for all 30 patients.

The median RMSE (IQR) between estimated and reference ISL was 6.9 mm (3.3 mm) (Fig. 8).

## Discussion

Most previous studies focused only on Adam’s forward bending test and static postures, whereas our study also investigated sequences dynamically captured during forward bending and lateral bending commonly used in clinical practice. These dynamic assessments are often used to evaluate mobility and offer advantages over static postures by providing insights into spinal flexibility and response to movement. We investigated how these dynamic postures influence the estimation of Cobb angle and internal spinal alignment (ISL) while standing upright. Incorporating dynamic evaluations could improve the accuracy of scoliosis assessment, offering a more comprehensive understanding of the deformity’s behavior and aiding in better treatment planning.

The comparisons of the overall estimate of the pCA (eCA) with the radiographic Cobb angle of the primary curve (rCA) from the clinical gold standard produced Pearson correlation coefficients within the range of 0.65 to 0.93 reported in the literature and median absolute errors comparable to the range of 4.7° to 7.5° reported in the literature^7,8,18–20^. Most previous studies focused on the estimation of the Cobb angle of primary curve (pCA); however, our comparison of estimated ISL with its reference is comparable to the range of 7.1 mm to 12 mm reported in the literature^21–23^. Our comparison of estimated SPLs and their reference for lateral bending produced very accurate results that are below the range of 2.84 mm to 5.8 mm reported in the literature^9,11,24^. However, because our results are for training data rather than test data, they overestimate the actual performance. Our continuing data collection will soon allow us to use estimated SPLs for all approaches and evaluate the RMSE more accurately with test data.

We use manually selected markers for all postures and movements and manually drawn SPLs for all movements and postures except lateral bending. This is a limitation because our approaches are not yet fully automated.

Our model for spinal alignment is limited to a simple line through the centroids of vertebral bodies. We have already developed a patient-specific 3D model that includes individual vertebrae from marker locations, SPL, and ISL. This is the focus of an upcoming publication.

The results presented here are for data captured from only 30 patients, which introduces bias in the regression results. This is also reflected in the wide confidence intervals, indicating that the true correlation could be lower than reported. A larger dataset would narrow these intervals, providing more precise and reliable estimates of model performance. Additionally, the limited sample size restricts parameter tuning, as proper fine-tuning with cross-validation requires more data to ensure robustness and prevent overfitting. Expanding the dataset will enable systematic optimization and improve the accuracy of our method.

Furthermore, the patients had a mean BMI of 20 kg/m^2^ with a standard deviation of 3 kg/m^2^, which falls within normal weight. Future work needs to investigate whether our approaches also work for underweight, overweight, and obese patients. It remains unclear how well our approaches generalize to these groups. A larger and more diverse dataset will enable us to assess performance across different BMI categories and provide more specific accuracy estimates for each subgroup.

To address these limitations, our continuing data collection will focus on increasing both the number and diversity of patients, enabling rigorous internal validation through cross-validation and external validation on independent datasets.

We have introduced approaches for which results show potential. Our publicly available platform supports further research to improve back-shape-to-spine approaches. The platform can be easily modified to test and improve various asymmetry approaches and is not limited to estimating pCA but is also capable of estimating ISL.

## Conclusion

We have presented noninvasive and nonionizing approaches to estimate the Cobb angle of the primary curve (pCA) and internal spinal alignment (ISL) in patients with adolescent idiopathic scoliosis. The approaches use back shape asymmetries captured with optical 3D scanning during upright standing, Adam’s forward bending test, bending forward, and lateral bending. The results obtained were comparable to the literature. The code of our unique platform is publicly available (see Data Availability Statement) to facilitate further improvement of back-shape-to-spine approaches and the eventual establishment of optical 3D surface scanning for scoliosis screening in clinical practice.

## Data Availability

The MATLAB code presented is openly available on GitHub at https://github.com/mkaisereth/PCdareSoftware including data.
